# Effect of selenium nanoparticles on biological and morphofunctional parameters of barley seeds (*Hordéum vulgáre* L.)

**DOI:** 10.1038/s41598-023-33581-6

**Published:** 2023-04-20

**Authors:** Andrey Ashotovich Nagdalian, Andrey Vladimirovich Blinov, Shahida Anusha Siddiqui, Alexey Alekseevich Gvozdenko, Alexey Borisovich Golik, David Guramievich Maglakelidze, Igor Vladimirovich Rzhepakovsky, Maxim Yurievich Kukharuk, Sergey Ivanovich Piskov, Maksim Borisovich Rebezov, Mohd Asif Shah

**Affiliations:** 1 North Caucasus Federal University, 1, Pushkin Street, 355017 Stavropol, Russia; 2 Campus Straubing for Biotechnology and Sustainability, Technical University of Munich, Essigberg 3, 94315 Straubing, Germany; 3 German Institute of Food Technologies (DIL e.v.), Prof.-Von-Klitzing-Straße 7, 49610 Quakenbrück, Germany; 4 Department of Scientific Research, V. M. Gorbatov Federal Research Center for Food Systems, Moscow, Russia; 5 Department of Economics, Kabridahar University, Kabridahar, Post Box 250, Somali Ethiopia; 6 Division of Research and Development, Lovely Professional University, Phagwara, Punjab India; 7 School of Business, Woxsen University, Hyderabad, Telangana 502345 India

**Keywords:** Biotechnology, Nanoscience and technology

## Abstract

The purpose of this work was to study the effect of selenium nanoparticles (Se NPs) on the biological and morphofunctional parameters of barley seeds (*Hordéum vulgáre* L.) We used seeds of *Hordéum vulgáre* L. with reduced morphofunctional characteristics. For the experiment, Se NPs were synthesized and stabilized with didecyldimethylammonium chloride. It was found that Se NPs have a spherical shape and a diameter of about 50 nm. According to dynamic light scattering data, the average hydrodynamic radius of the particles was 28 ± 8 nm. It is observed that the nanoparticles have a positive ζ-potential (+ 27.3 mV). For the experiment, we treated *Hordéum vulgáre* L. seeds with Se NPs (1, 5, 10 and 20 mg/L). The experiment showed that treatment of *Hordéum vulgáre* L. seeds with Se NPs has the best effect on the length of roots and sprout at concentration of 5 mg/L and on the number and thickness of roots at 10 mg/L. Germinability and germination energy of *Hordéum vulgáre* L. seeds were higher in group treated with 5 mg/L Se NPs. Analysis of macrophotographs of samples, histological sections of roots and 3D visualization of seeds by microcomputing tomography confirmed the best effect at 5 mg/L Se NPs. Moreover, no local destructions were detected at concentrations > 5 mg/L, which is most likely due to the inhibition of regulatory and catalytic processes in the germinating seeds. the treatment of *Hordéum vulgáre* L. seeds with > 5 mg/L Se NPs caused significant stress, coupled with intensive formation of reactive oxygen species, leading to a reorientation of root system growth towards thickening. Based on the results obtained, it was concluded that Se NPs at concentrations > 5 mg/L had a toxic effect. The treatment of barley seeds with 5% Se NPs showed maximum efficiency in the experiment, which allows us to further consider Se NPs as a stimulator for the growth and development of crop seeds under stress and reduced morphofunctional characteristics.

## Introduction

The problem of grain crops’ resistance to stress factors has become extremely relevant in recent years due to the increasing negative impact of the environment on the formation of productivity^1,2^. The study of plant reactions to periodically occurring stressful situations is necessary to develop ways to reduce their negative impact. The same reaction of plants to different stress factors was found when studying the effects of soil drought^3–5^, high temperatures^6–8^, and salinity^9–11^. The primary non-specific response of plants to the damaging effects of heavy metals, low temperatures, UV radiation, herbicides and flooding is also the formation of reactive oxygen species^12–18^. The accumulation of reactive oxygen species and the development of oxidative stress is one of the early effects of stress on plants and can manifest itself even at the pre-sowing stage, especially during long grain storage^19–21^.

The analysis of the current state of the problems of sustainability and the realization of the adaptive potential of plants indicate the presence of a common mechanism to counter various stressors, the functioning of which reduces energy costs for the formation of specialized adaptation mechanisms. Numerous studies of plant adaptation to various stressors have established the presence of a single mechanism for the development of damage and protection under any abiotic stress^22,23^.

Increasing the adaptability of plants to adverse environmental factors can be achieved by using growth regulators. Their use enhances metabolic processes, increases plant resistance to stressful conditions, which ultimately increases the yield of field crops and improves the quality of agricultural products^24^. In this area, a number of works are aimed at identifying additional exogenous stimulating factors in the initial period of plant growth and development^25–28^. Recently, nanoparticles of essential trace elements have been increasingly considered in agriculture as growth stimulators increasing adaptive potential of crops^29,30^, among which various modifications of selenium nanoparticles (Se NPs) attract attention^31,32^.

The supply of plants with selenium significantly determines their physiological state, which opens up the possibility by regulating the mineral nutrition of plants to directly affect certain aspects of metabolism, which may be useful in the development of resistance to adverse environmental factors. A large amount of information has been accumulated describing the participation of selenium in various physiological processes^33–35^. Most scientific research is aimed at identifying the functions of selenium in seeds of agricultural crops, specifically, its growth-stimulating value. Wang et al. discovered genes that regulate selenium metabolism in plant organisms, which confirms the need for the trace element selenium for plants and the importance of further studying the role of selenium in plant physiology^36^. Hu et al. studied Se NPs uptake mechanisms in wheat^37^. The authors found that addition of aquaporins inhibitor causes 92.5% and 93.4% inhibition of chemosynthesized Se NPs and biosynthesized Se NPs absorption by wheat roots. Absorption rate depended on nanoparticle size, nature of Se NPs and inhibitor application. Wang et al. obtained Se-enriched rice by direct application of Se NPs, selenite or selenate on rice seedlings^38^. Adhikary et al. found that seed priming with the combination of Se NPs and Zn NPs is a viable option for the risk mitigation in direct-seeded rice (*Oryza sativa* L.) and provides better germination, growth, and yield of rice^39^. Similar results were obtained by Galić et al. when studying the physiological response of soybean seedlings enriched with Se NPs to osmotic stress^40^. Zahedi et al. found that spraying solutions containing SiO_2_, Se and Se/SiO_2_ nanoparticles (50 and 100 mg/L) improves the growth and yield parameters of strawberry plants grown under normal conditions and drought stress conditions (38 °C)^41^.

In general, a number of studies have shown the role of selenium in enhancing the adaptive potential of plants^42^. Selenium plays a protective role by exhibiting an antioxidant effect^43^. Li et al. found that Se NPs activate the plant antioxidant system, resulting in significant increases in the content and activity of antioxidant enzymes in the ascorbic acid-glutathione cycle, which helped minimize pesticide induced oxidative damage in tea leaves^44^. El Saadony et al. obtained Se NPs suspension produced by *Lactobacillus acidophilus* and studied its antiradical activity^45^. It was observed that biosynthesized Se NPs suspension (150 µg/mL) was significantly better absorbed DPPḢ and ABTS + radicals compared to tert-butyl hydroquinone (TBHQ). The authors pointed out that antioxidant and antifungal activity of obtained biosynthesized Se NPs provided reducing crown and root rot diseases incidence and counteracting drought and heat stress in wheat. The same results on antioxidant activity of Se NPs were declared in other works^46–48^. It is important to note that the antioxidant potential of Se NPs is due to the small size^49^ and the electron-donating ability of bioactive compounds on the nanoparticles’ surface^50,51^.

It was found that selenium is able to significantly increase the stress resistance of seeds in air and soil drought, hypoxia and in conditions of increased salinity of the environment^52–54^. In drought conditions, selenium has a protective effect on wheat seedlings^55^, slows down biomass reduction and increases wheat yield^56^.

Selenium affects the activity of antioxidant enzymes of plants and the level of peroxidation under stress. Soldatov and Raschetova state that treatment of wheat seedlings with 10^–4^ and 10^–5^% sodium selenate solution leads to decrease of the amount of peroxidation products in hypothermia conditions by 40–42%^57^. The authors also noted a 66–68% decrease in the activity of superoxide dismutase, a 22–38% decrease in the activity of glutathione reductase and a strong activation of proteinase activity. Prudnikov and Krivushina found that the trace element selenium under simulated conditions of hyperthermic effect on the leaves of red currant activates the work of the antioxidant enzyme catalase by 2.2 times^58^. The discovered effect slowed down the process of peroxide damage of membrane lipids under stress. Khan et al. observed that selenium affects the formation of ethylene and reduces cadmium-induced oxidative stress, improving the production of proline and glutathione in wheat^59^.

In laboratory and field experiments, some researchers confirmed the positive effect of selenium-containing compounds on the stress resistance of grain crops to adverse environmental factors: drought, soil salinity, soil acidity and hyperthermia^60–63^, as well as the positive effect of selenium-containing fertilizers on the productivity of grain crops^64,65^. The effect of selenium on plant growth and development depends on the forms of selenium-containing fertilizers used and the methods of their application^66^.

The effect of Se NPs on the adaptive potential of plants under stress has been studied on a limited number of species and requires further research to give an objective scientific assessment of these effects. The role of selenium in the regulation of plant life also remains insufficiently studied. For example, our previous studies showed that selenium nanoparticles with a concentration of 4.65 µg/mL provide better germination and germination energy of barley seeds (*Hordéum vulgáre* L.)^67^. At the same time, with an increase in the Se NPs concentration, a suppression of seed growth indicators was observed. Several researchers have also reported similar results. According to Chernikova et al. when stimulating the germination of corn seeds, the optimal concentration of Se NPs is 3.7 mg/L, whereas a high concentration has a toxic effect^68^. The toxic effect of Se NPs on crop seeds at high concentrations was declared by other researchers^13,69–75^.

Prins et al. studied the effect of selenate on the reproductive functions of accumulating and hyperaccumulating species of Se^59^. The authors observed that the germination rate decreased with increasing concentrations of selenate in brown mustard. Experiment of Garousi et al. showed toxicity in both sunflower and maize plants by application Se NPs with high concentration (> 10 mg/L)^70^. El Mehdawi et al. observed reduction and inhibition of the germination of *A. thaliana* species sown in soil close to Se hyperaccumulator species due to their apparent ability of concentrate Se, in which the concentration of 10 mg/L of Se inhibited seed germination by 50%^71^. According to Lapaz et al. application of 20 mg/L of Se NPs decreased the biomass of shoot and roots of cowpea seedlings^75^. The authors observed that total sugars and sucrose concentration in cowpea seedlings increased in response to Se concentration exposure which was explained as the stress caused by the Se concentrations. Similar results were obtained by Silva et al. treated cowpea plants with high concentrations of Se preparation under field conditions^72^. Mostofa et al. found that excessive Se NPs caused phytotoxic effects on rice plants by inducing chlorosis, reducing sugar, protein and antioxidant contents, and exacerbating oxidative stress and methylglyoxal toxicity^73^. At the same time the authors suggested that accumulation levels of Se, proline and glutathione could be considered as efficient biomarkers to indicate degrees of Se-induced phytotoxicity in rice, and perhaps in other crops.

Thus, studies conducted in recent decades have shown that selenium is a biogenic element necessary for plants. The effect of selenium under stress obviously has a multifunctional value that requires additional research. Many questions covering the mechanisms of action of these elements, their role in stress tolerance, remain unresolved. Another important question is the toxicity of Se NPs to crop seeds. Therefore, study of the effect of selenium on growth, development and physiological processes in seeds of agricultural crops is relevant and has a significant scientific soundness. The purpose of our research was to study the effect of Se NPs on the biological and morphofunctional parameters of barley seeds (*Hordéum vulgáre* L.).

## Materials and methods

*Hordéum vulgáre* L. seeds were used in this experiment. The seeds were stored for more than 10 years, with reduced morphofunctional characteristics. Selected seeds were not subjected to additional processing before.

Under laboratory conditions, the effect of Se NPs on the sowing qualities and biological properties of seeds was studied, determining germinability and germination energy, as well as morphological parameters such as the number and roots, length of sprout and roots. To determine the nature of morphofunctional changes, microstructural analysis of seeds using histology and microcomputing tomography (µCT) methods was carried out.

The experiment was performed with accordance to all the ISTA (2006) germination requirements. Reagent grade chemicals and grade A glassware were used in the present study. Conductivity of distilled water used was less than 1 µS/cm. The experiments were carried out in threefold biological and fivefold analytical repetition.

### Selenium nanoparticles synthesis

Synthesis of Se NPs was carried out by the method of chemical reduction of a selenium-containing precursor in an aqueous medium in the presence of stabilizers. For the synthesis we used didecyldimethylammonium chloride (GC ETS, St. Petersburg, Russia) as a stabilizer, selenic acid (INTRERHIM, St. Petersburg, Russia) as a selenium-containing precursor and ascorbic acid (Lenreactive, St. Petersburg, Russia) as a reducing agent. The choice of stabilizer, precursor and reducing agent and their characteristics are justified and described in our previous work^66^. According to our protocol, synthesis of Se NPs stabilized with didecyldimethylammonium chloride (DDAC) was carried out in three stages. At the first stage, solutions of DDAC were prepared. For this we dissolved from 0.68 to 5.24 g of DDAC in 100 cm^3^ of 0.036 M selenic acid. At the second stage, 0.088 M ascorbic acid solution was prepared by dissolving 773.8 mg of ascorbic acid in 50 cm^3^ of double distilled water. At the third stage, a solution of ascorbic acid was added drop by drop to a solution of selenic acid and stabilizer with intensive stirring and the resulting sample was mixed for 10 min.

### Investigation of selenium nanoparticles

The microstructure of Se NPs was studied using a Carl Zeiss Libra 120M (Carl Zeiss NTS GmbH, Oberkochen, Germany) transmission electron microscope. Se NPs were applied by ultrasonic dispersion of a solution of the test sample and water in a ratio of 1:1 on copper grids with a carbon base. The magnitude of the accelerating voltage of the thermal emission gun TEM Libra 120M (Carl Zeiss NTS GmbH, Oberkochen, Germany) was 120 kV.

The determination of the average hydrodynamic radius of the particles was carried out by the dynamic light scattering (DLS) method on a Photocor-Complex instrument (Antek-97, Moscow, Russia). Processing of the results was carried out using the DynaLS software.

To study the obtained samples, IR spectrometer FSM 1201 with Fourier transform (Ifraspec, Saints Petersburg, Russia) was used. Research parameters:

- measurement range—from 400 to 4000 cm^−1^.
- measurement step—1 cm^−1^.

Investigation of the ζ-potential was carried out by acoustic and electroacoustic spectroscopy on the DT-1202 installation (Dispersion Technology Inc., Bedford Hills, NY, USA).

The molecular modelling was carried out in the IQmol molecular editor (SIBUR, Moscow, Russia). The quantum-chemical calculations of the models were carried out using the QChem software (SIBUR, Moscow, Russia) with the following parameters: Calculation—Energy, method—B3LYP, Basis—6-31G*, Convergence—4, Force field—Chemical.

### Seeds preparation

Seeds (50 units per group) were placed in a Petri dish on filter paper under optimal humidification conditions at a temperature of 20 °C for 7 days. Ratio of liquid phase and seeds was 4:5. The liquid phase we used is as follows:

- distilled water (Sample A);
- 1 mg/L Se NPs solution (Sample B);
- 5 mg/L Se NPs solution (Sample C);
- 10 mg/L Se NPs solution (Sample D);
- 20 mg/l Se NPs solution (Sample E).

### Investigation of biological parameters of seeds

The germination energy and germination ability were evaluated according to the ISTA (2006) standard. Germination energy, linear dimensions of sprouts and roots and number of roots were determined after 3 days, and seeds germinability after 7 days.

Macrophotography of seeds was carried out using a laboratory binocular microscope Axio ZOOM.V16 (Carl Zeiss Microscopy, Oberkochen, Germany) at a magnification of 20× with image fixation using a specialized AxioCam MRc5 camera (Carl Zeiss Microscopy, Oberkochen, Germany) and the Zen software (Carl Zeiss Microscopy, Oberkochen, Germany).

### Histological analysis of seeds

To carry out histological study of germinated seeds, we prepared histological micropreparations of root system and sprout from each group. Using the microtome MZP-01 Technom with the microtome cooler OMT-28-02E (KB-Technom, Yekaterinburg, Russia), histological sections of roots and sprout were made at a distance of 5 mm from the seed. The average thickness of the cut was 0.05 mm. Micropreparations were stained with phloroglucinol (Lenreactive, St. Petersburg, Russia) in the presence of hydrochloric acid (Lenreactive, St. Petersburg, Russia) and were processed on a Levenhuk D870T microscope (Levenhuk, Tampa, FL, USA) with a Levenhuk C510 digital camera at magnification 100×. Micrographs were processed in the Levenhuk ToupView 3.7 program (Levenhuk, Tampa, FL, USA). As a result, we processed micrographs of roots and sprout histological sections and calculated the average values of conducting bundle area.

### Microcomputing tomography of seeds

3D visualization of samples was carried out using a Skyscan 1176 X-ray microcomputing tomography system (Bruker, Billerica, MA, USA). The X-ray voltage and current was 65 kV and 380 μA, respectively, filter Al 1 mm. Scanning of the whole seeds volume was carried out using 8.77 μm pixel size as resolution, taking about 60 min. The scan protocol included rotation through 180° at 0.3° as rotation step, an exposure time of 1175 ms per frame, frame averaging—2. 3D reconstruction of samples was created using the reconstruction NRecon 1.7.1.0 software (Bruker, Billerica, MA, USA). For tomographic reconstruction the following settings were used: no smoothing, ring artifact correction = 14 and beam hardening correction = 51%. CTAn 1.13.11.0 software (Bruker MicroCT, Kontich, Belgium) was used for the quantitative analyses upon the reconstructed structure. Global thresholding was then used to segment the grayscale images into binary black/white images to facilitate the quantitative analysis and 3D visualization of the structure. Visualization of µCT results was obtained by program software DataViewer and CTVox (Bruker MicroCT, Kontich, Belgium).

### Statistical analysis

All parameters obtained were submitted to one-way analysis of variance (ANOVA) and Student’s T-test (p < 0.05) through the statistical package STATISTICA for Windows (Statsoft, Tulsa, USA). Microsoft Excel 2010 and Origin software were used for histograms and graphs creation based on the data obtained.

## Results and discussion

### Investigation of selenium nanoparticles

**Figure 1:**
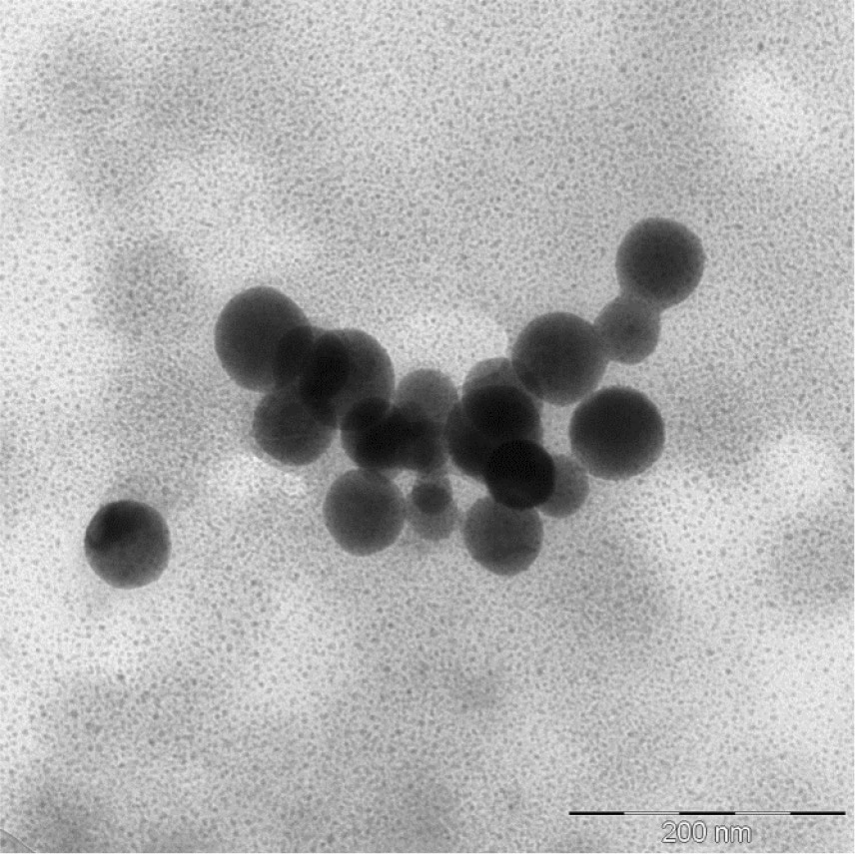
TEM micrograph of Se NPs stabilized with DDAC.

As a result of transmission electron microscopy of prepared Se NPs samples we obtained micrographs, one of which is shown in Fig. 1.

**Figure 2:**
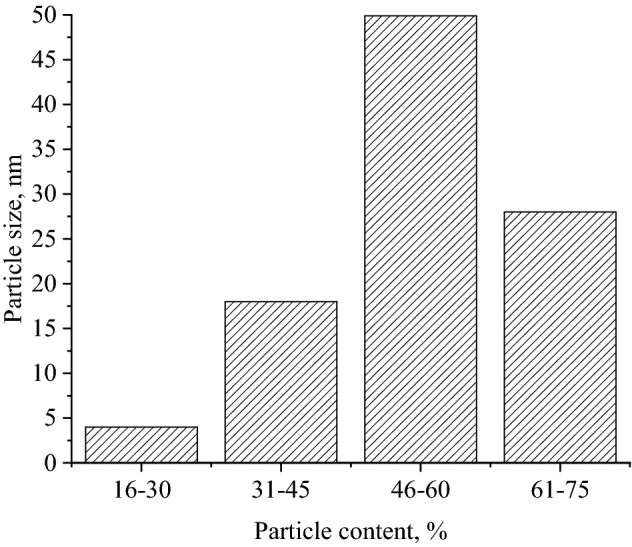
Histogram of the size distribution of Se NPs stabilized with DDAC.

According to Fig. 1, Se NPs had a spherical shape with evenly covered DDAC coatings and were in a stable condition. Based on the obtained TEM micrographs we formed a histogram of the size distribution of Se NPs (Fig. 2).

Thus, according to the data obtained, it was established that Se NPs synthesized in the presence of DDAC have moderate polydispersity with an average diameter of about 50 nm.

**Figure 3:**
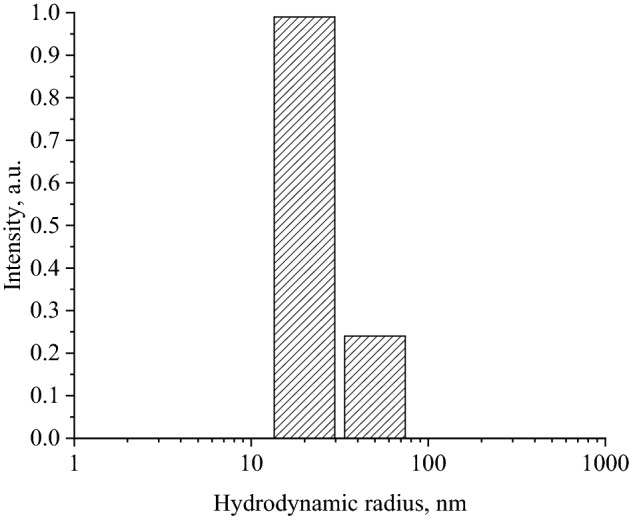
Histogram of the distribution of the hydrodynamic radius of Se NPs.

At the next stage, the average hydrodynamic radius of the obtained Se NPs was investigated by DLS. The histogram of the distribution of the hydrodynamic radius of Se NPs is shown in Fig. 3.

Analysis of the resulting histogram showed that the average hydrodynamic radius of Se NPs was 28 ± 8 nm, which corresponds to the TEM results and is similar to the data described in the previous work^76^.

**Figure 4:**
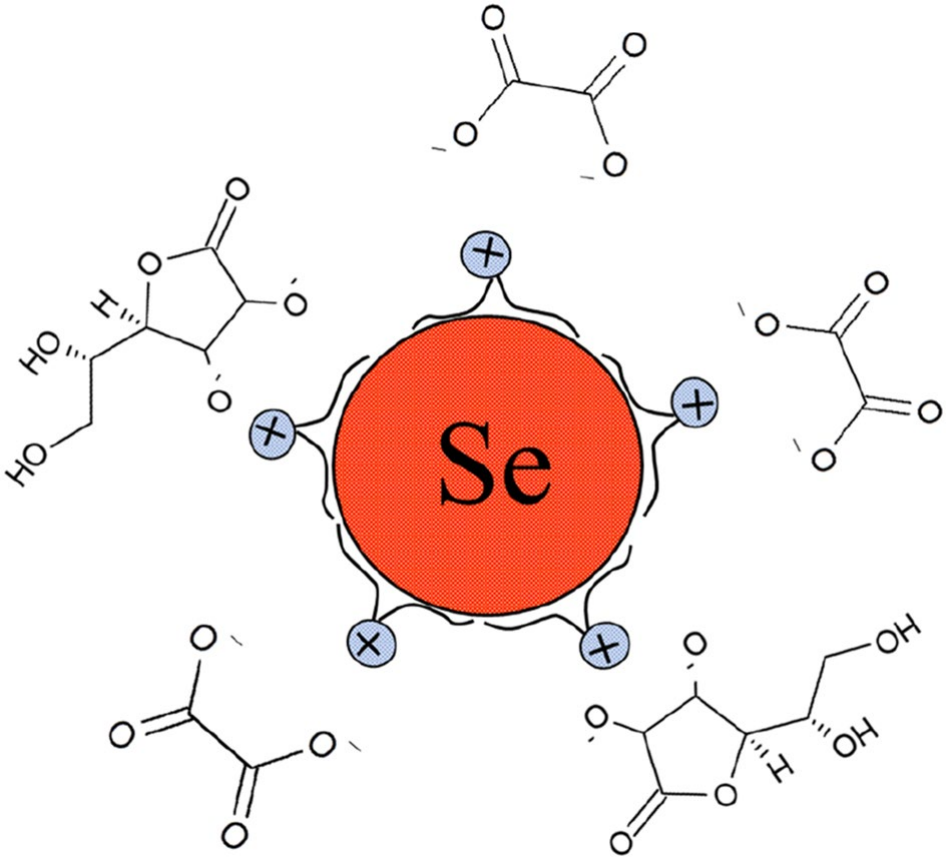
Structure of micelles of Se NPs stabilized with DDAC.

Measurements of the ζ-potential revealed that Se NPs have a positive electrokinetic potential equal to + 27.3 mV. Based on the results obtained, we assumed the structure of micelles for Se NPs stabilized with DDAC shown in Fig. 4.

The surface of Se NPs is hydrophobic, hence the DDAC molecule will attach to the surface of the nanoparticles with a hydrophobic tail. According to the Fajans-Paneth-Hahn Law, the potential-forming layer will be formed by positively charged particles^77^. The layer of counterions is formed by oxalic acid anions, which were formed as a result of oxidation of ascorbic acid molecules.

**Figure 5:**
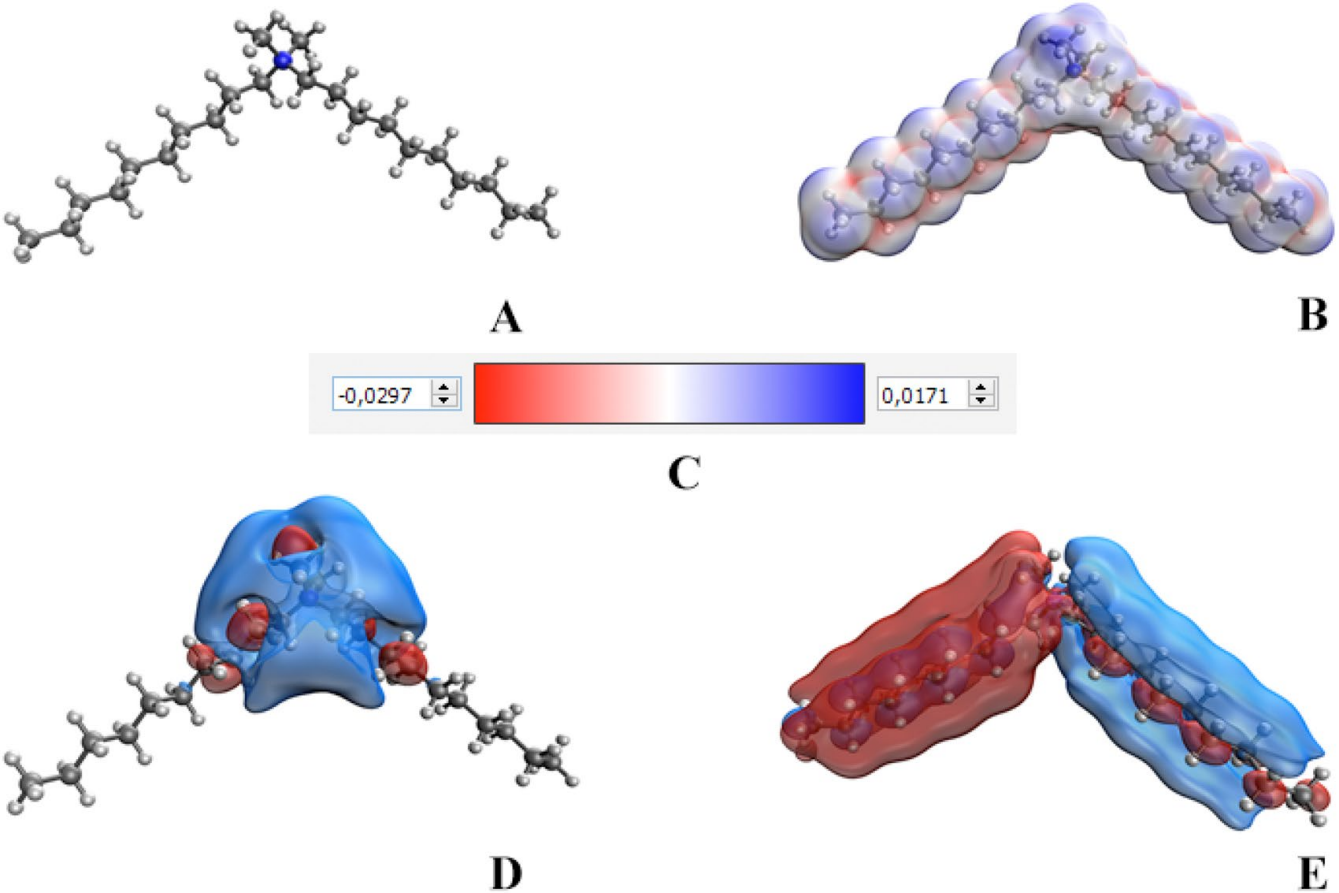
Results of quantum chemical modeling of the DDAC: molecular model (**A**), electron density distribution (**B**), electron density distribution gradient (**C**), highest occupied molecular orbital HOMO (**D**), lowest unoccupied molecular orbital LUMO (**E**).

**Figure 6:**
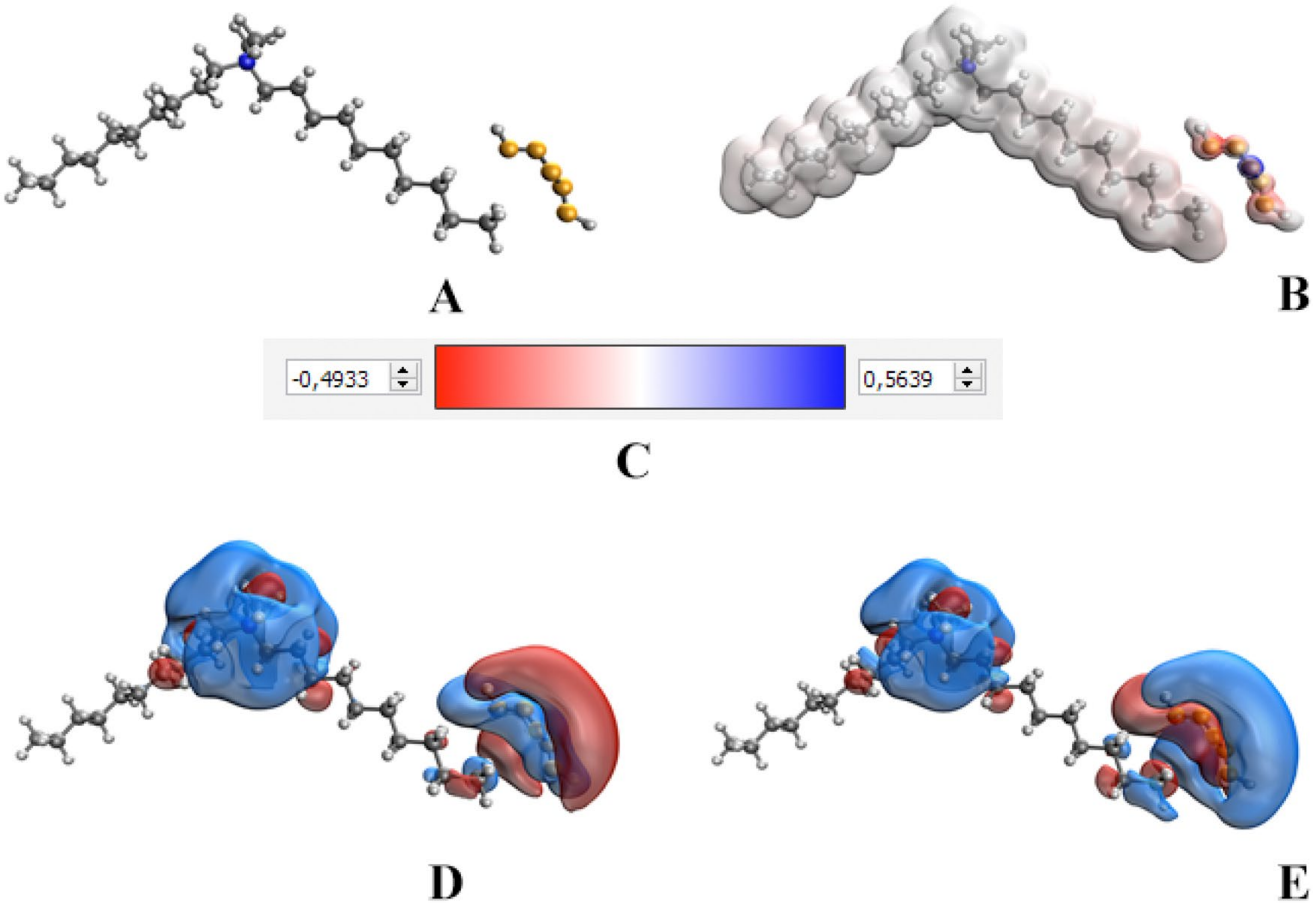
Results of quantum chemical modeling of Se NPs–DDAC: molecular complex model (**A**), electron density distribution (**B**), electron density distribution gradient (**C**), highest occupied molecular orbital HOMO (**D**), lowest unoccupied molecular orbital LUMO (**E**).

At the next stage of the research, quantum chemical modeling of the DDAC molecule and the molecular complex Se NPs – DDAC was carried out. The resulting models are shown in Figs. 5, 6.

As a result of computer quantum chemical modeling, it was found that the total energy of the DDAC molecule is − 921.833 kcal/mol, and the energy of the Se NPs–DDAC molecule complex is − 12,913.626 kcal/mol. The energy of the highest occupied molecular orbital (0.000) and the lowest unoccupied molecular orbital (0.008) for molecular complex Se NPs–DDAC was determined. With the chemical hardness coefficient n = 0.004, our calculations confirmed stability of developed Se NPs–DDAC molecular complex. As a result of quantum chemical modeling, we have established that the molecular complex Se NPs–DDAC is energetically advantageous and stable^78,79^.

**Figure 7:**
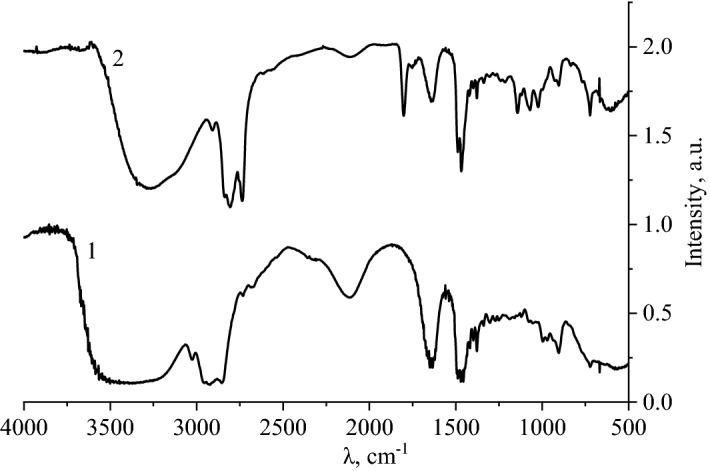
IR spectrum of selenium nanoparticles stabilized by DDAC: 1—DDAC; 2—Se NPs stabilized with DDAC.

At the next stage of research, the obtained samples were examined by IR spectroscopy. The resulting IR spectrum is shown in Fig. 7.

Analysis of the IR spectrum of DDAC showed that in the range from 2800 to 3300 cm^−1^, the presence of oscillation bands characteristic for CH_2_ group (2854–2930 cm^−1^) and for CH_3_ group (2957–3028 cm^−1^) is observed. In the range from 1200 to 1800 cm^−1^, there is the presence of symmetrical oscillations of CH_2_ group, which correspond to the bands 1377 cm^–1^ and 1651 cm^−1^, and symmetrical oscillations of CH^3^ group, which correspond to the oscillation band in the range 1271–1340 cm^−1^. Also in this range there are oscillation bands characteristic of the NH^+^ group (1470 and 1494 cm^−1^). In the range from 500 to 1200 cm^−1^, the presence of oscillation bands characteristic for CH_2_ group is observed^80–82^.

Analysis of the IR spectrum of Se NPs stabilized with DDAC showed that in the range from 2800 to 3300 cm^−1^, the presence of oscillation bands characteristic for CH_2_ group (2731, 2855 and 2926 cm^−1^), for CH_3_ group (2955 and 3021 cm^−1^) and CH group (3397 and 3462 cm^−1^). In the range from 1200 to 1800 cm^−1^, the presence of symmetrical oscillations characteristic for CH_2_ group is observed, which correspond to the intensity drop region of 1339 cm^−1^, 1377 cm^−1^ and 1634 cm^−1^, respectively. There are also fluctuations characteristic for CH^3^ group (1209 cm^−1^, 1240 cm^−1^, 1269 cm^−1^, 1302 cm^−1^, 1751 cm^−1^ and 1800 cm^−1^), for NH^+^ group (1466 cm^−1^ and 1489 cm^−1^). In the range from 500 to 1200 cm^−1^, there is the presence of bond fluctuations characteristic for CH^2^ group (835 cm^–1^, 905 cm^−1^, 930 cm^−1^, 997 cm^−1^, 1020 cm^−1^ and 1068 cm^−1^) and for CH group (1121 cm^−1^ and 1146 cm^−1^). The IR spectrum of Se NPs contains vibrations characteristic of metallic Se (600 cm^−1^ and 665 cm^−1^) and for the asymmetric Se–O bond (725 cm^−1^)^83–85^.

### Investigation of the effect of Se NPs on seeds germination rates

**Figure 8:**
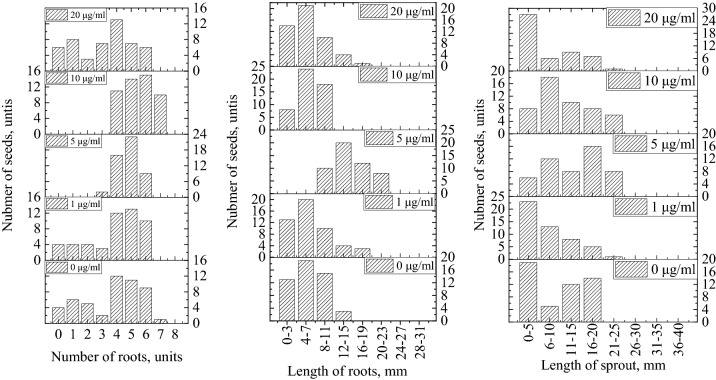
Histogram of the distribution of the number of roots, length of roots and length of the sprout of *Hordéum vulgáre* L. seeds depending on Se NPs concentration.

The results of calculating the number and length of roots and length of sprout of *Hordéum vulgáre* L. seeds for easier perception, we presented this in the form of histograms (Fig. 8).

Based on the data presented in Fig. 8, the average values of number of roots, length of roots and length of the sprout are calculated and summarized in Table 1.

**Table 1:** The average value of number of roots, length of roots and length of the sprout in the experimental groups.

Experimental group	Number of roots	Length of roots	Length of sprout
Group A	3.72	6	5.46
Group B	3.88	6.66	6.94
Group C	4.72	14.36	14.44
Group D	5.44	6.52	11.6
Group E	3.16	5.96	6.32

The significance of the data presented in Table 1 was confirmed by ANOVA (Table 2).

**Table 2:** ANOVA of data on number of roots, length of roots and length of the sprout of *Hordéum vulgáre* L. seeds.

Source of variation	SS	df	MS	F	F_crit_	p-value
Number of roots						
Between groups	161.06	4	40.26	15.7966	2.4085	< 0.05
Within groups	624.48	245	2.55			
Total	785.54	249				
Length of roots						
Between groups	2627.36	4	656.84	44.3916	2.4085	< 0.05
Within groups	3625.14	245	14.80			
Total	6252.50	249				
Length of the sprout						
Between groups	3014.98	4	753.75	16.5466	2.4085	< 0.05
Within groups	11,160.44	245	45.55			
Total	14,175.42	249				

*SS* sum of squares, *dF* degree of freedom, *MS* mean sum of squares, *F* F-value, *F*_*crit*_*.* critical F-value.

From the data given in Table 1, it can be observed that in all groups F > Fcrit. Therefore, the null hypothesis is rejected. ANOVA showed that considered indicators in *Hordéum vulgáre* L. seeds significantly prevailed in groups C and D with a determination coefficient (η^2^) of 20.5% (number of roots), 42% (length of roots) and 21.3% (length of sprout). Therefore, to determine the significance of the difference between groups C and D, a Student's T-test was applied with the selected significance level α = 0.05 (Tables S1–S3, Supplementary). According to the data obtained, the average length of roots was significantly higher in group C (14.44 mm) than in group D (6.52 mm). The average length of sprout in group C (14.36 mm) was significantly higher than in group D (11.6 mm). However, seeds in group D had significantly more roots (5.44) than seeds in group C (4.72). Thus, we have identified the first trend according to which the processing of *Hordéum vulgáre* L. seeds with Se NPs has the best effect on the length of roots and sprout at concentration of 5 mg/L and on the number of roots at 10 mg/L. The revealed trend approximately corresponds to the results described in other works^57,67,68^.

**Figure 9:**
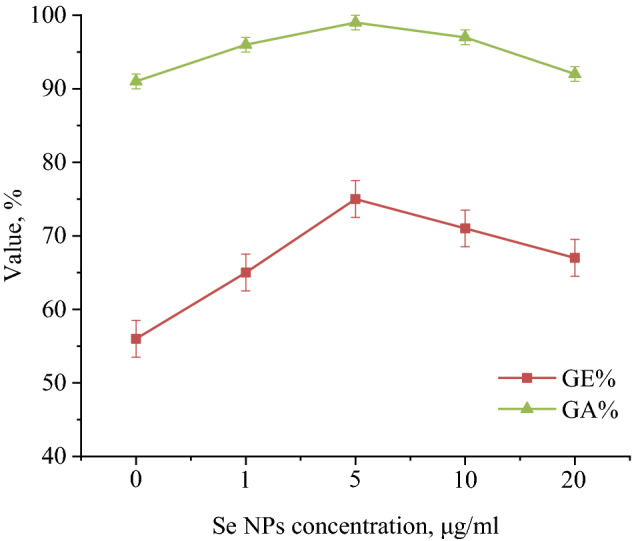
Dependence of germination energy (GE) and germinability (GA) of *Hordéum vulgáre* L. seeds on Se NPs concentration.

Other important indicators of the growth and development of seeds are germinability and germination energy. Figure 9 shows the calculated trend of germinability and germination energy of *Hordéum vulgáre* L. seeds.

These calculations showed that Se NPs have a significant effect on the increase in GE and GA. The maximum GE was obtained at 5 mg/L Se NPs—76%, which is more than the control sample by 19%. Treatment of *Hordéum vulgáre* L. seeds with Se NPs at concentration of more than 5 mg/L contributed to an increase in the GE value to 66–71%. However, this effect in samples with > 5 mg/L Se NPs was leveled compared to the control sample upon further observation. GA in the control group was 91%, which corresponds to the data provided in ISTA (2006). The treatment increased the GA in the experimental groups to 95–99%. The maximum germination was established at 5 mg/L Se NPs while higher concentration inhibited GA and GE. The same trend was observed by other authors in studies on *Arabidopsis thaliana*^71^ and *Vigna unguiculata*^75^. Thus, the data obtained indicates the non-linearity of the influence of different concentrations of Se NPs on the morphological parameters of seeds during germination.

**Figure 10:**
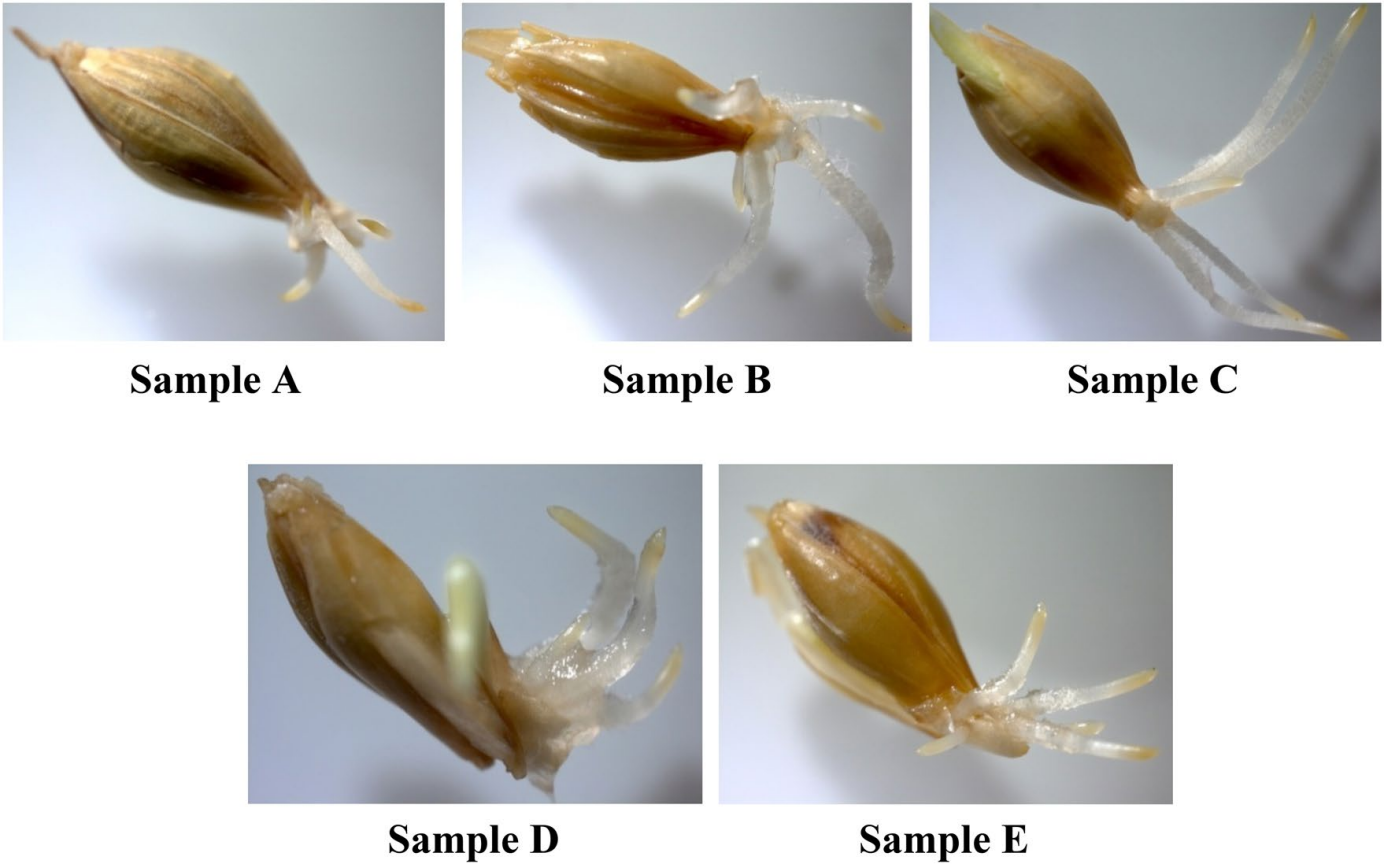
Macrophotographs of *Hordéum vulgáre* L. seeds on the third day of germination (magnification ×20).

Photographs (Fig. S1, Supplementary) and macrophotographs (Fig. 10) of *Hordéum vulgáre* L. seeds on the third day of germination also clearly demonstrate the changes occurring in the samples depending on the Se NPs concertation.

The analysis of macrophotographs showed that the most intensive growth of sprout and formation of root system occurred at 5 mg/L Se NPs (C). However, the largest root thickness we observed in Fig. 10 (D), which confirms the nonlinear nature of the effect of Se NPs treatment on the morphology of seeds during germination, which was also declared by Chernikova et al.^68^.

There can be many reasons for the nonlinearity of the Se NPs influence factor on *Hordéum vulgáre* L. seeds. A high concentration of Se NPS can inhibit phytohormones and seed enzymes, affect the antioxidant potential, contribute to the destruction of morphological microstructures, or affect the throughput of membranes and nutrient channels^86–88^. For comprehensive assessment and understanding of these processes we carried out a histological examination of seeds and analyzed 2D micrographs of histological sections of control and experimental samples. The average area of the conducting bundles of the root system and sprout are presented in Table 3.

**Table 3:** Average area of conducting bundles of the root system and sprout, p < 0.05.

Sample	Area of conducting bundles S, µm^2^	
	Root system	Sprout
A	280,004	110,795
B	287,218	119,332
C	292,452	123,205
D	298,939	121,122
E	273,453	117,030

According to the data presented in Table 3 the largest area of conducting bundles was in Samples C and D, which correlates with the results of biological and morphological study of seeds. It is important to note that the thickness of the roots was greater in Sample D, but the sprout was thicker in Sample C, which indicates the need to study the root system and sprouts in experimental samples in more detail to understand the mechanism of Se NPs influence on *Hordéum vulgáre* L. seeds in a better way.

**Figure 11:**
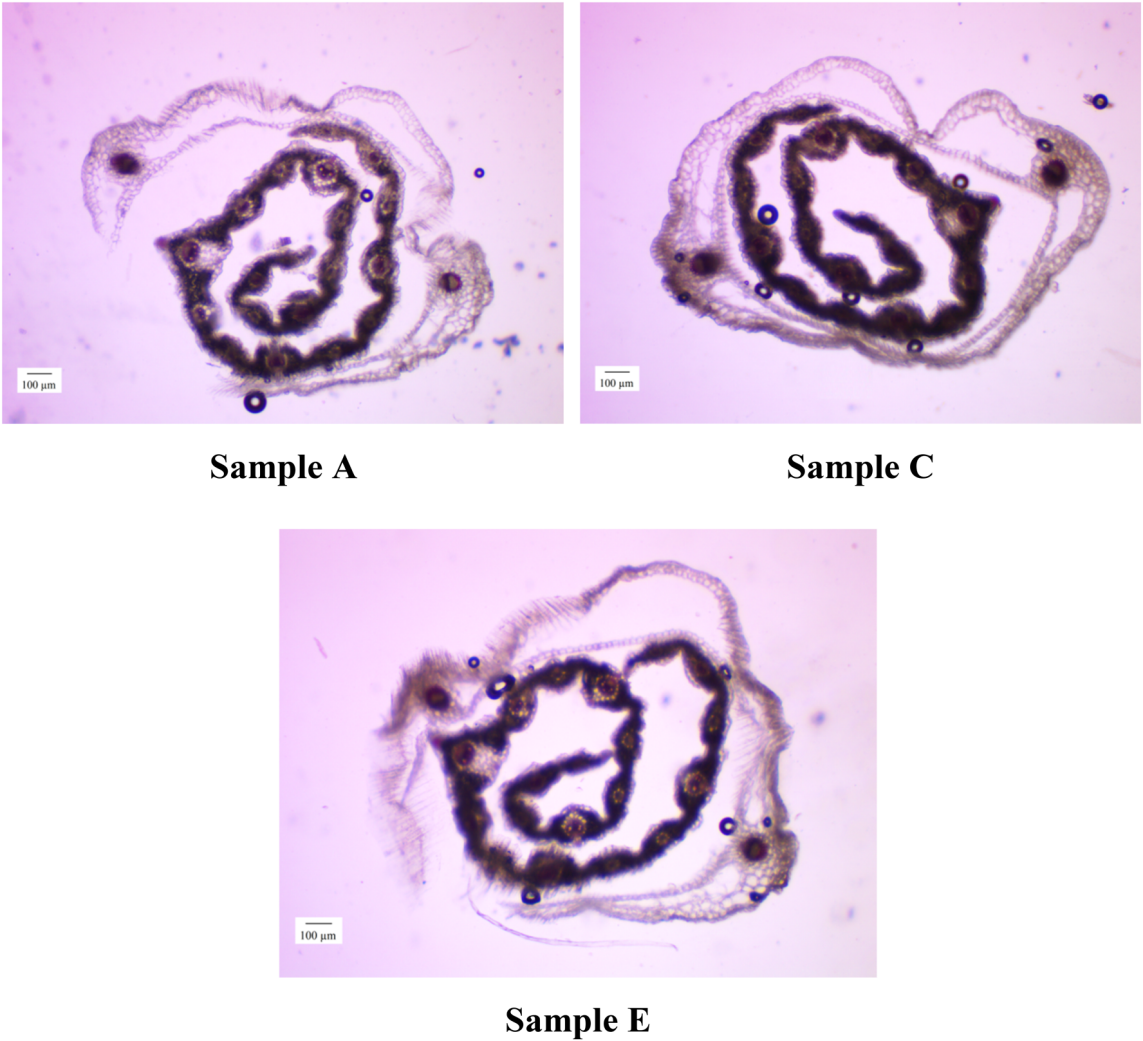
Typical 2D micrographs of the crosswise histological section of the seed sprout of Samples A, C and E at magnification ×100.

Figure 11 shows typical 2D micrographs of a crosswise histological section of the seed rout of Samples A, C, E (samples B and D had intermediate characteristics).

The analysis of the crosswise histological sections presented in Fig. 11 allowed us to draw the appropriate conclusions for each sample.

Sample A is characterized by a large number of vacuoles of water in the area of cap and the germinating first leaf. There are groups of cambial cells, as well as phloem cells, chlorenchyma and sclerenchyma, weakly stained with phloroglucinol. The sprout has a spiral tubular structure with well-defined supporting skeleton with the development of vascular-fibrous collateral bundles. Deposits of starch grains are weakly expressed; there are small amount of swollen starch grains having an unbranched structure.

Sample C is stained in the area of cap and the germinating first leaf. There is a moderate amount of water vacuoles. Cambial cells are swollen. There is the formation of adventitious roots, phloem cells, chlorenchyma and sclerenchyma, stained with phloroglucinol. The sprout has a spiral tubular structure with a medium pronounced supporting skeleton. Starch grains tend to swell and mainly have a branched structure.

Sample E is stained in the area of cap and the germinating first leaf. Very low concentration of water vacuoles is observed. Cambial cells are weakly shown; the core is swollen; the sclerenchyma of the pericycle and epidermal cells are poorly colored; chlorenchyma is formed, but stained with low intensity. The sprout has a spiral tubular structure with a moderately pronounced supporting skeleton in the core. There is an intensive deposition of starch grains, which tend to swell and have a predominantly branched structure.

Summarizing the data obtained, we can conclude that there was a more intensive use of endosperm reserve nutrients in Samples C and E. The optimal distribution of moisture concentration and the most intensive activation of growth energy is determined in Sample C. Thus, the study of crosswise histological sections of the sprout of seed samples showed that 5 mg/L Se NPs contribute to the greatest intensity of seed germination. At the same time, no local destructions were detected in Sample E compared to Sample C, which indicates that the decrease in the effectiveness of Se NPs at concentrations > 5 mg/L is most likely due to inhibition of regulatory and catalytic processes in the germinating seed^89,90^.

**Figure 12:**
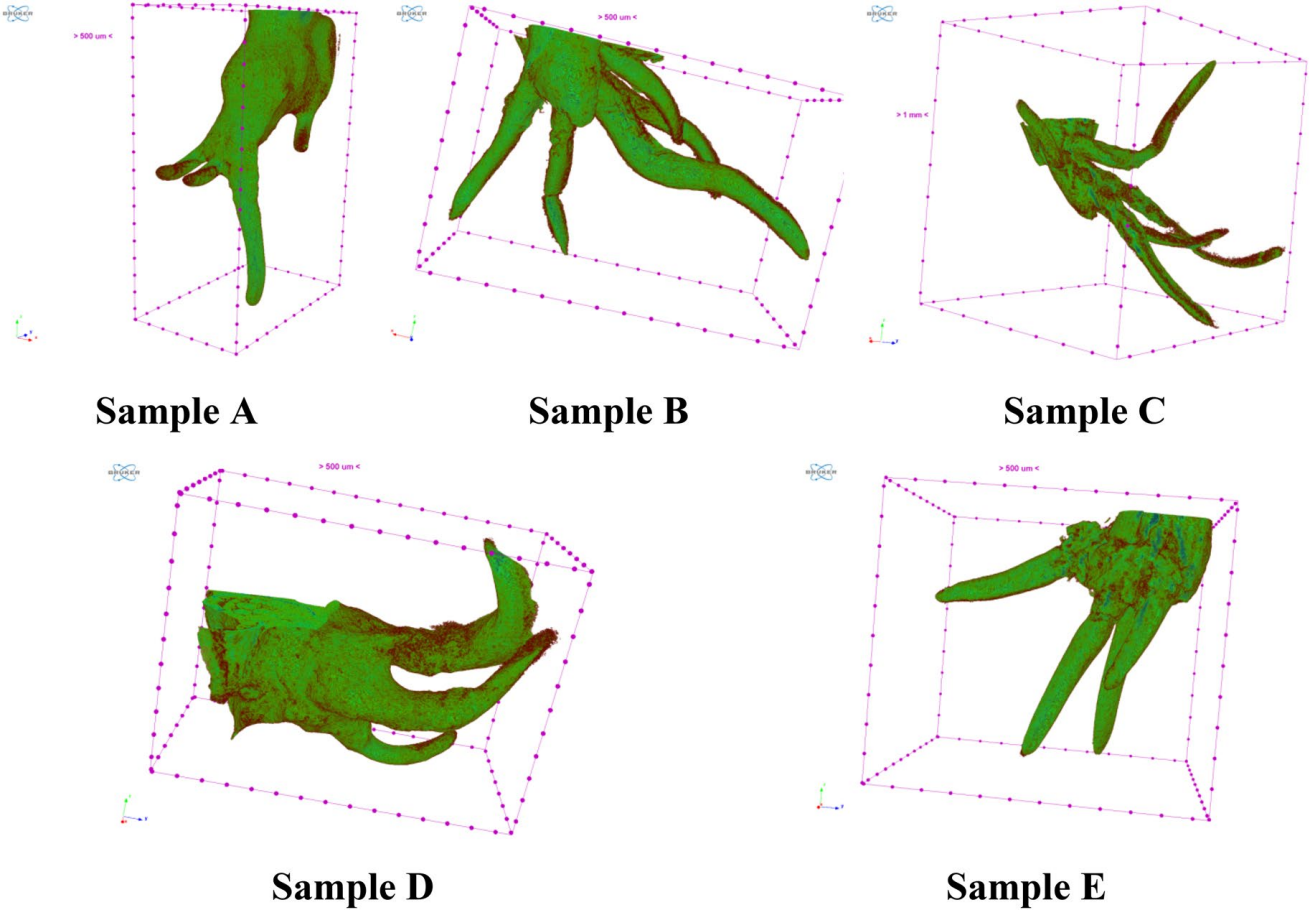
3D visualization of roots of seed samples by µCT.

**Figure 13:**
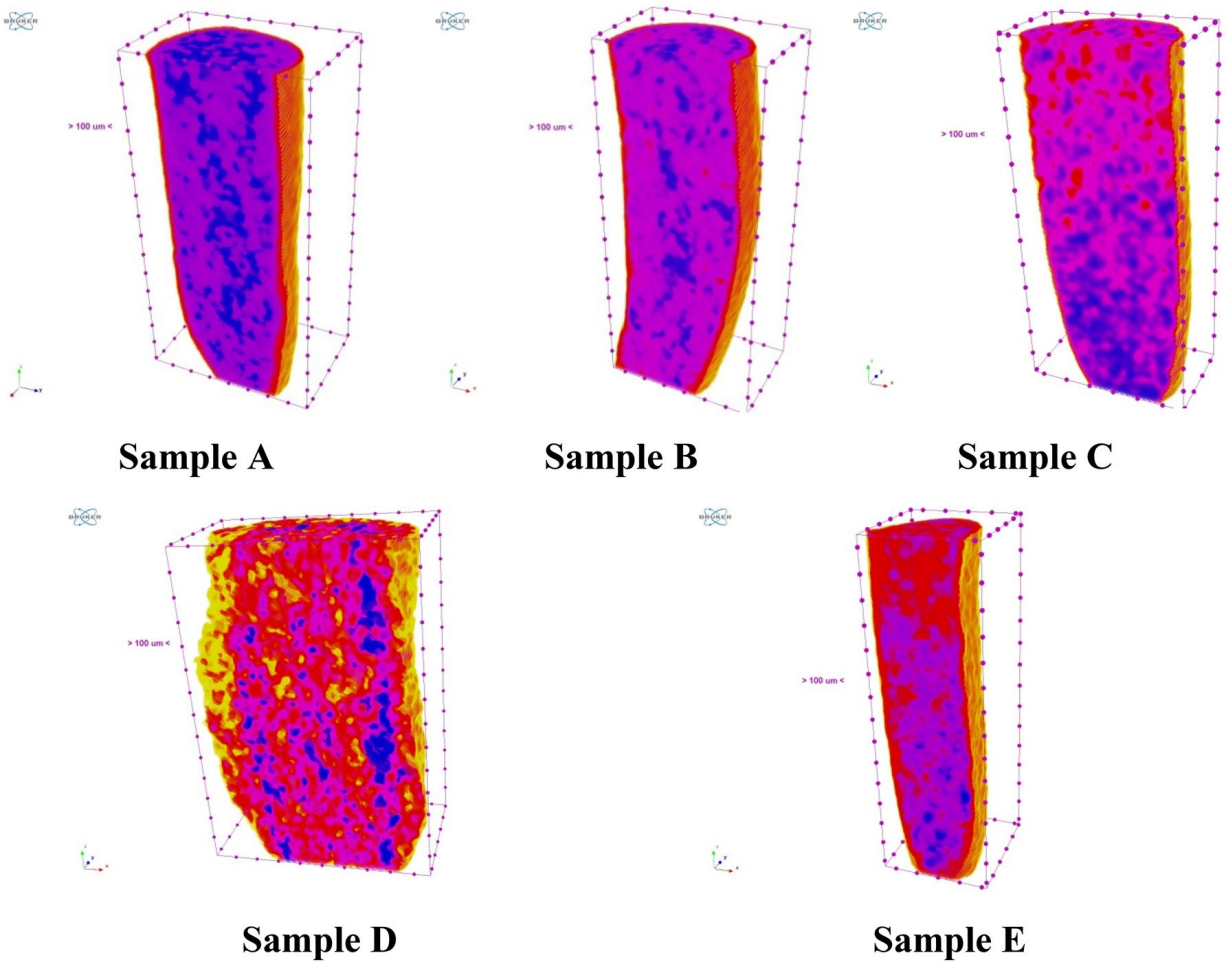
3D visualization with color identification of the X-ray density of the roots structure.

More detailed analysis of seed samples microstructure was carried out using µCT. We obtained 3D images of sprout and roots (Fig. 12), further processing of which made it possible to make cross sections with an assessment of geometric parameters and X-ray density (Fig. 13).

Analysis of Fig. 12 showed that the average thickness of the roots was 508 µm in Sample A, 530 µm in Sample B, 541 µm in Sample C, 640 µm in Sample D and 487 µm in Sample E, which approximately corresponds to results of histological analysis. The greatest length of roots was observed in Sample C (12.7 mm), which also confirms the highest efficiency of Se NPs at a concentration of 5 mg/L.

Research work studying the activity of various forms of selenium confirm the positive effect of selenium and selenium-containing compounds on plant roots growth in the early stages of germination, as shown on lentils, rice, potatoes, bittermelon and tomatoes, etc.^91–95^. Similar results were obtained by Kiryushina et al. in the study of the effect of sodium selenite on the formation of roots system of barley seeds^96^. Selenium at a concentration of 0.0005% contributed to an increase in root length by 31%, and coleoptile length by 22% in comparison with the control. The authors observed that with a higher concentration of selenium in sodium selenite (0.001%) there was a tendency to decrease the length of the coleoptile (by 17%). Thus, barley seeds are sensitive to selenium, especially at high concentrations (> 0.0005%). These data are also consistent with the data we studied about the possible toxic effect of selenium on agricultural crops during pre-sowing treatment^13,74,75,97^. It is important to understand the mechanism of structural changes at high concentrations of selenium. For this we analyzed X-ray density of experimental seeds. The X-ray density can be identified on the objects shown in Fig. 13 and Supplementary (Figs. S2–S6). Color density differentiation: X-ray density increases from red to blue through purple.

According to the specified parameters of color differentiation, dense structures (blue) were identified, which are evenly distributed in the control sample, but strongly shift towards the end of the root depending on the concentration of Se NPs. Hence, we detected a structural anomaly in the experimental samples. To explain the nature of the established microstructural changes caused by Se NPs, an in-depth analysis of the available scientific literature in this field is necessary.

There is a possibility that selenium can enhance the proteolytic activity of papain enzymes and lead to the release of tryptophan, which is a precursor of the hormone heteroauxin (indolylacetic acid)^72,98^. The increase in the thickness and length of roots may indicate that Se NPs can indirectly enhance mitotic activity due to the synthesis of auxin (heteroauxin) at the root tip^99,100^. It is believed that free (physiologically active) auxin is a signal necessary for vascular differentiation^101,102^. Since the bound hormone is not involved in vascular differentiation^103,104^, we believe that the disorientation of structural elements in the experimental samples occurs as a result of the transformation of auxin from a bound form to a free one.

Recently, it has been widely believed that auxin in combination with active oxygen forms are the main regulators of plant development under various stresses^19,105^. At the same time, it is known that the concentration of auxin may decrease due to a reduction in hormone biosynthesis with an increase in the content of reactive oxygen species (ROS), conjugation of the hormone, or due to its direct oxidation when oxidative stress occurs^106,107^. The accumulation of ROS in seeds induced by stress in the form of a high concentration of Se NPs can directly affect auxin metabolism by affecting the genes responsible for hormone signaling^108,109^.

Timilsina et al. observed that prolonged exposure to stress leads to changes in growth mechanisms, reductions in cell divisions and increased lateral growth^110^. These processes are carried out due to interactions between ROS and auxin. This means that when seeds were treated with > 5 mg/L Se NPs, the samples were subjected to significant stress, coupled with intensive formation of ROS, which led to a reorientation of root system growth towards thickening. Treatment of seeds with 20 mg/L Se NPs apparently turned out to be excessive, which is most likely due to partial suppression of the hormone auxin with abundant formation of ROS, which also contributed to the oxidation of many biosynthesis processes. Since Sample E is significantly weaker from a biological and morphological point of view than Sample A, treatment of *Hordéum vulgáre* L. seeds with 20 mg/L Se NPs is biologically and economically ineffective. Moreover, based on the results indicating a structural reorientation of the germination of *Hordéum vulgáre* L. seeds in Samples D and suppression of morphofunctional characteristics of Sample E, we declare a logical conclusion that > 5 mg/L Se NPs have a toxic effect on barley. Treatment with 5 mg/L Se NPs in our work, on the contrary, showed maximum efficiency on *Hordéum vulgáre* L. seeds, which allows us to further consider Se NPs as a stimulator of growth and development of agricultural seeds under stress and reduced morphofunctional characteristics. At the same time, scientific interest is aroused by further study of the mechanisms of multidirectional stimulation of the growth of agricultural seeds during the treatment of Se NPs in the optimal concentration range with detailed study of enzymes, phytohormonal status and antioxidant properties of treated seeds.

## Conclusion

Our work proved that the effect of Se NPs on seeds of agricultural crops has a multifunctional value. It was found that Se NPs have a spherical shape and a diameter of about 50 nm. According to dynamic light scattering data, the average hydrodynamic radius of the particles was 28 ± 8 nm. It is shown that the nanoparticles have a positive ζ-potential (+ 27.3 mV). The data obtained indicate the non-linearity of the influence of different concentrations of Se NPs on the morphological parameters of seeds during germination. The experiments showed that treatment of *Hordéum vulgáre* L. seeds with Se NPs has the best effect on the length of roots and sprout at concentration of 5 mg/L and on the number and thickness of roots at 10 mg/L. Germinability and germination energy of *Hordéum vulgáre* L. seeds were higher in groups treated with 5 mg/L Se NPs. Histological studies also confirmed that the optimal distribution of moisture concentration and the most intense activation of germination energy was determined in a sample with 5 mg/L Se NPs. At concentrations > 5 mg/L, no local destructions were detected, which is most likely due to inhibition of regulatory and catalytic processes in the germinating seed. More detailed information about the microstructure of seed samples was obtained by microcomputing tomography. The cross section of roots showed that the average root thickness was 508 µm in the control sample, 530 µm at 1 mg/L Se NPs, 541 µm at 5 mg/L Se NPs, 640 µm at 10 mg/L Se NPs and 487 µm at 20 mg/L Se NPs, which approximately corresponds to the data obtained during histological analysis. The greatest length of root processes was observed in a sample with 5 mg/L Se NPs (12.7 mm), which also confirms the highest efficiency of Se NPs at a concentration of 5 mg/L.

Based on results of microcomputing tomography we suggested that high concentration of Se NPs can inhibit the phytohormones and enzymes of seeds, affect the antioxidant potential, contribute to the destruction of morphological microstructures or affect the throughput of membranes and nutrient channels. In our opinion, treatment with > 5 mg/L Se NPs causes significant stress in *Hordéum vulgáre* L. seeds, coupled with intensive formation of reactive oxygen species, which lead to a reorientation of root system growth towards thickening. Treatment of *Hordéum vulgáre* L. seeds with 20 mg/L Se NPs apparently turned out to be excessive. This is most likely due to partial suppression of the hormone auxin with abundant formation of reactive oxygen species, which also contributed to the oxidation of many biosynthesis processes. Since the obtained sample is significantly weaker from a biological and morphological point of view than the control sample, treatment of *Hordéum vulgáre* L. seeds with 20 mg/L Se NPs is ineffective from a biological and economic point of view.

Thus, the experiment showed that Se NPs can be considered further as a stimulator of growth and development of agricultural seeds under stress and reduced morphofunctional characteristics. At the same time, scientific interest is aroused by further study of the mechanisms of multidirectional stimulation of the growth of agricultural seeds during the treatment of Se NPs in the optimal concentration range with detailed study of enzymes, phytohormonal status and antioxidant properties of treated seeds.

## Supplementary Information

Supplementary Information.

## Data Availability

All raw data are available upon request from corresponding author.
