# Supplementary material for: Effect of selenium nanoparticles on biological and morphofunctional parameters of barley seeds (Hordéum vulgáre L.)
Source: Sci Rep. 2023 Apr 20;13:6453. doi: 10.1038/s41598-023-33581-6 (PMC10119286; doi:10.1038/s41598-023-33581-6)
Supplement: Supplementary file 1 — Supplementary Information. [file 41598_2023_33581_MOESM1_ESM.docx]

**Supplementary**

**Table S1.** Student’s T-test for number of roots between experimental groups

| Experimental group | Indicator | B | C | D | E |
| --- | --- | --- | --- | --- | --- |
| A | t | -0.4155 | -3.3512 | -5.4458 | 1.4318 |
|  | p-value | 0.6787 | 0.0011 | 3.82e-07 | 0.1554 |
| B | t |  | -2.9596 | -5.1638 | 1.8941 |
|  | p-value |  | 0.0039 | 1.27e-06 | 0.0612 |
| C | t |  |  | -4.0249 | 5.3437 |
|  | p-value |  |  | 1.12e-04 | 5.93e-07 |
| D | t |  |  |  | 7.3612 |
|  | p-value |  |  |  | 5.7e-11 |

**Table S2.** Student’s T-test for length of roots between experimental groups

| Experimental group | Indicator | B | C | D | E |
| --- | --- | --- | --- | --- | --- |
| A | t | -0.8094 | -11.198 | -0.7845 | 0.0507 |
|  | p-value | 0.4203 | 3.11e-19 | 0.4346 | 0.9597 |
| B | t |  | -9.3021 | 0.1859 | 0.8075 |
|  | p-value |  | 3.96e-15 | 0.8529 | 0.4213 |
| C | t |  |  | 11.5646 | 10.4685 |
|  | p-value |  |  | 5.09e-20 | 1.17e-17 |
| D | t |  |  |  | 0.7722 |
|  | p-value |  |  |  | 0.4418 |

**Table S3.** Student’s T-test for length of sprout between experimental groups

| Experimental group | Indicator | B | C | D | E |
| --- | --- | --- | --- | --- | --- |
| A | t | -1.1708 | -6.6761 | -4.5888 | -0.6062 |
|  | p-value | 0.2445 | 1.48e-09 | 1.32e-05 | 0.5458 |
| B | t |  | -5.8615 | -3.6632 | 0.457 |
|  | p-value |  | 6.18e-08 | 4.04e-04 | 0.6487 |
| C | t |  |  | 2.0997 | 5.6686 |
|  | p-value |  |  | 0.0383 | 1.45e-07 |
| D | t |  |  |  | 3.7031 |
|  | p-value |  |  |  | 3.52e-04 |


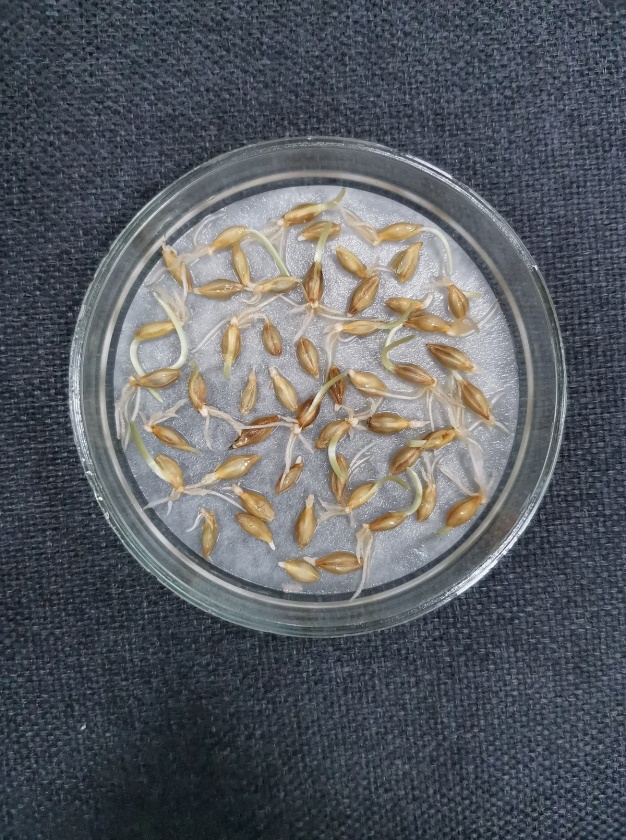

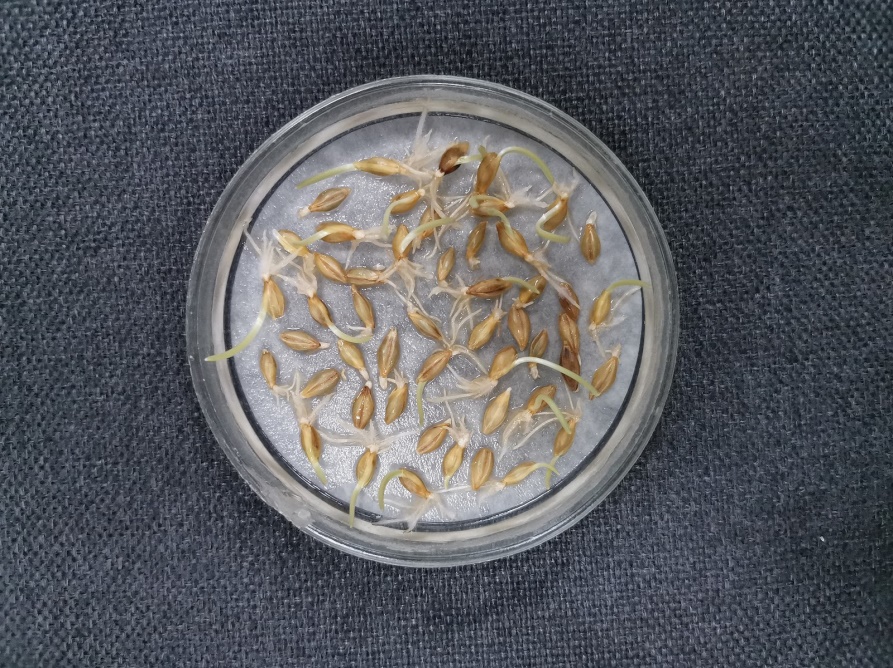


A B


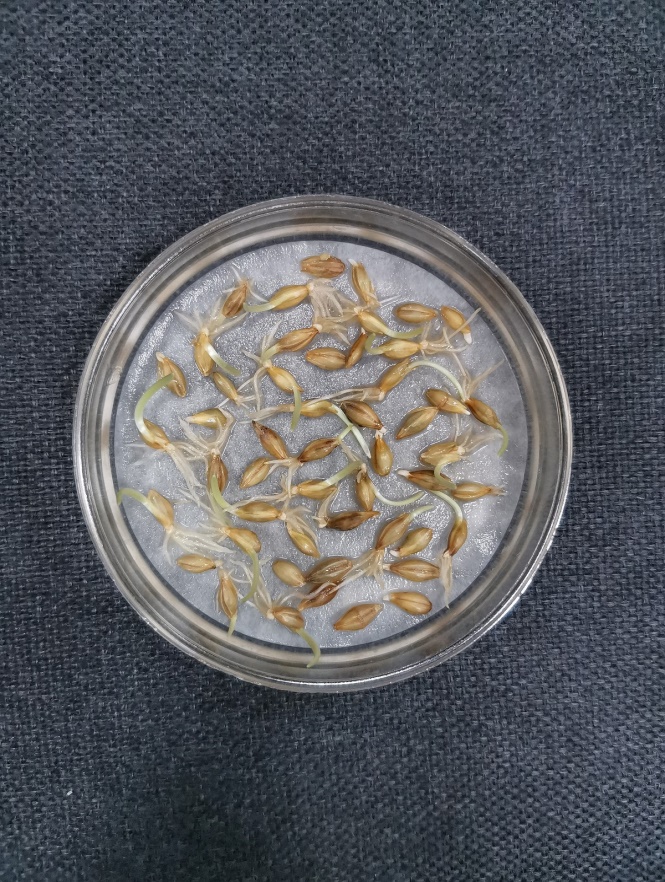

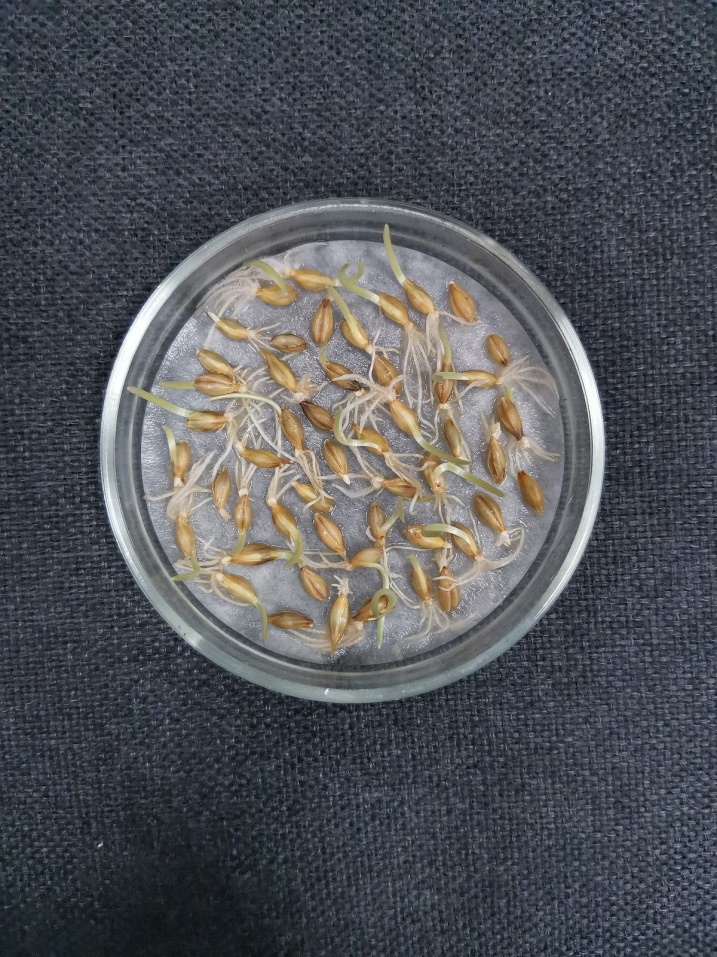


C D


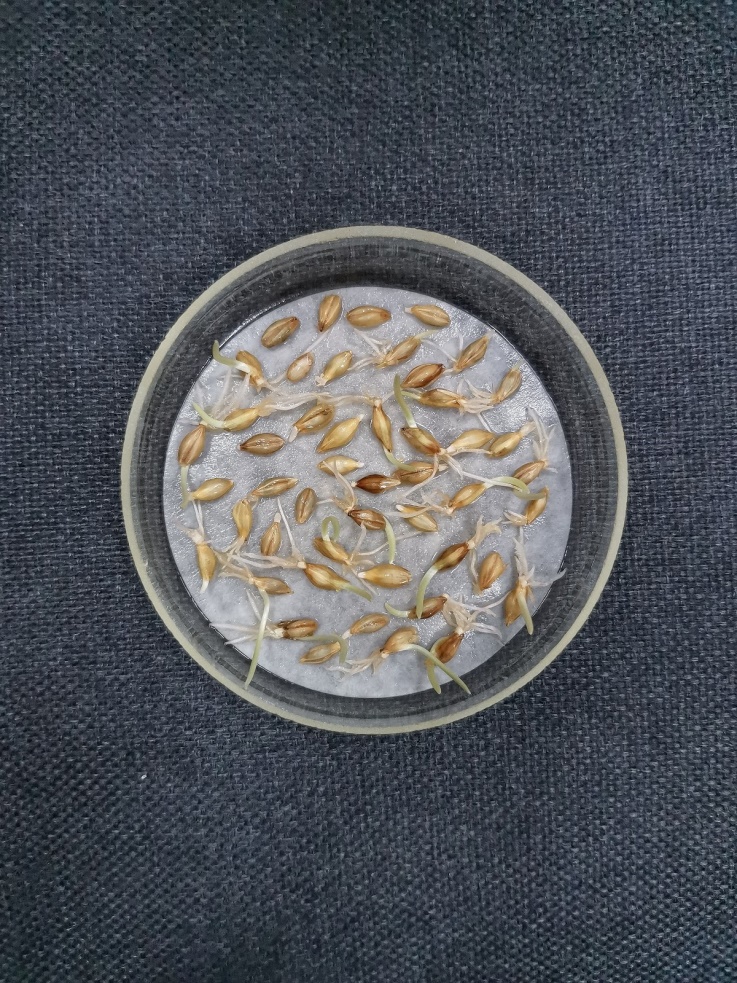


E

**Figure S1**. Photos of *Hordeum vulgare L*. seeds samples on the third day of germination. A – seed treated with distilled water (Sample A), B – seeds treated with 1 mg/L Se NPs solution (Sample B), C – seeds treated with 5 mg/L Se NPs solution (Sample C), D – seeds treated with 10 mg/L Se NPs solution (Sample D),

E – seeds treated with 20 mg/L Se NPs solution (Sample E).


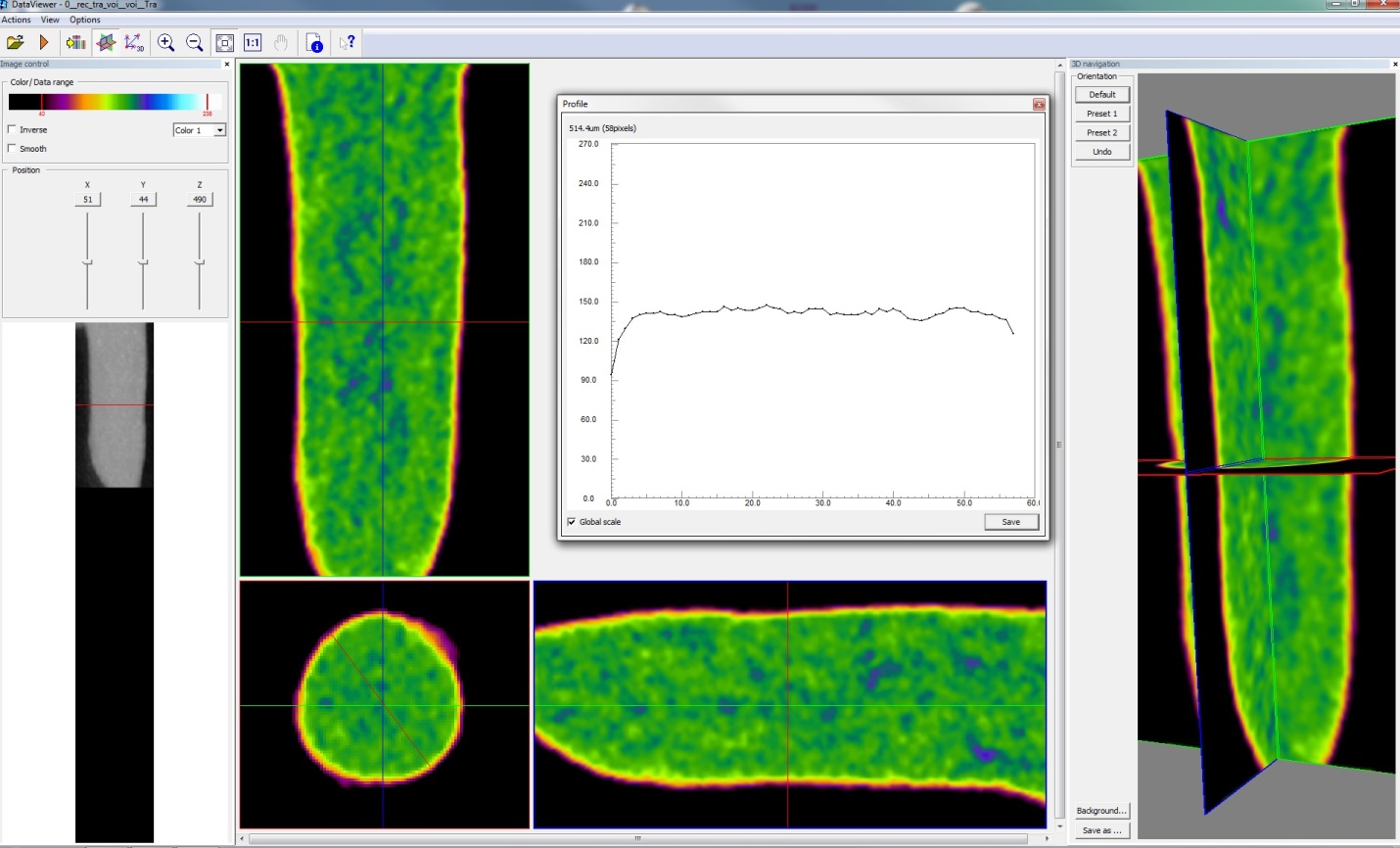


**Figure S2**. Analysis of X-ray contrast density and topology of the cross-section density of the root growth of sample А


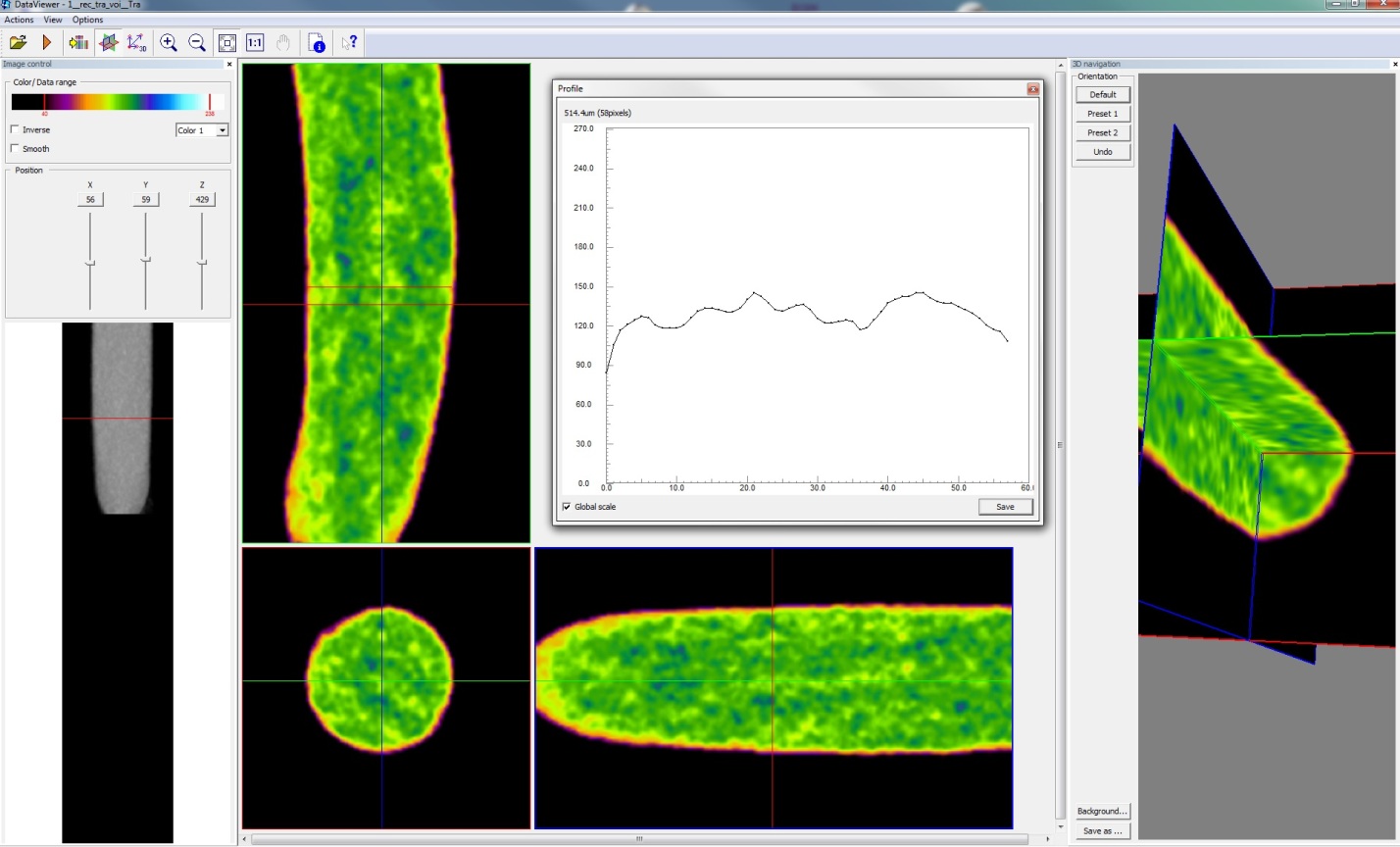


**Figure S3**. Analysis of X-ray contrast density and topology of the cross-section density of the root growth of sample B


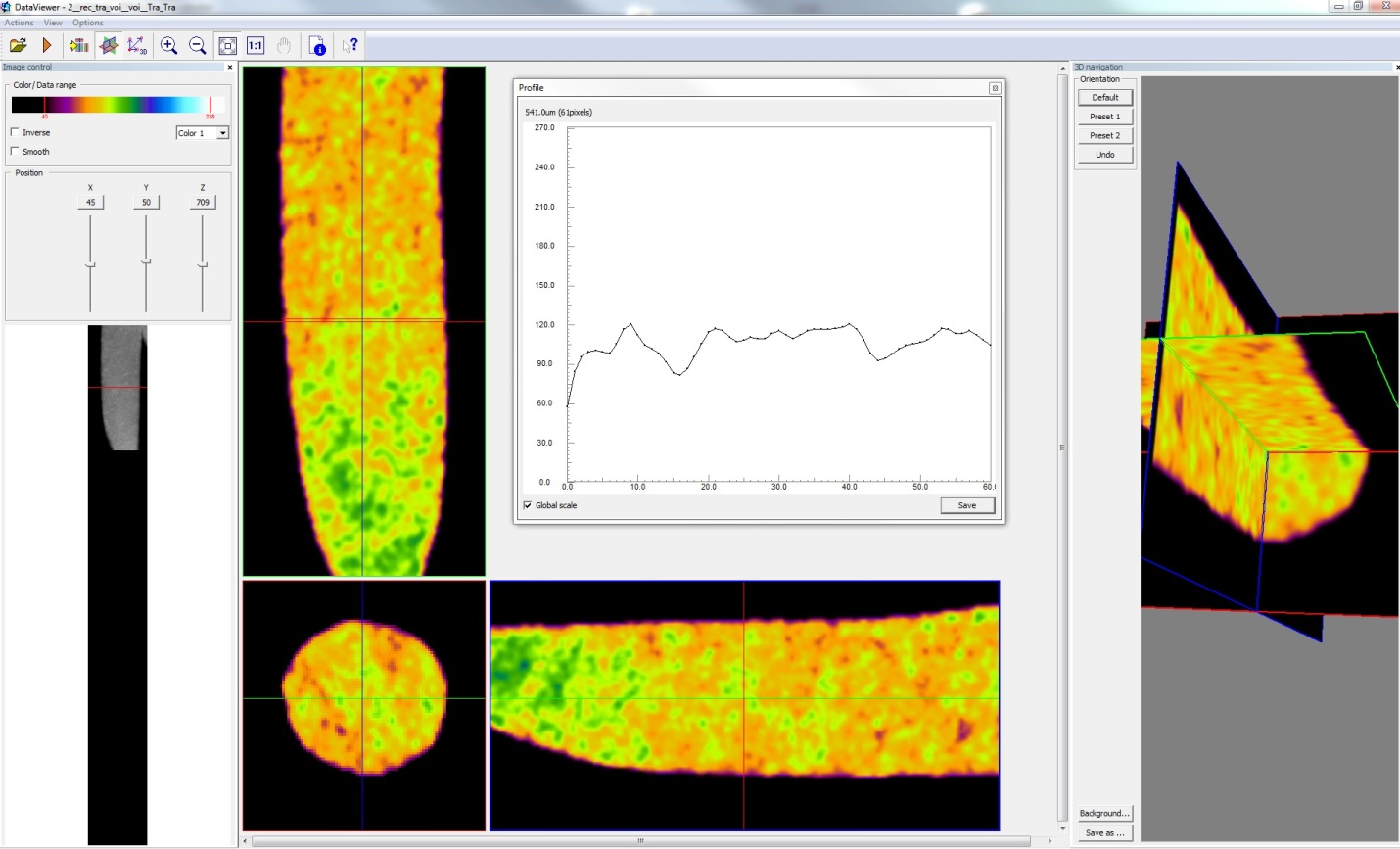


**Figure S4**. Analysis of X-ray contrast density and topology of the cross-section density of the root growth of sample C


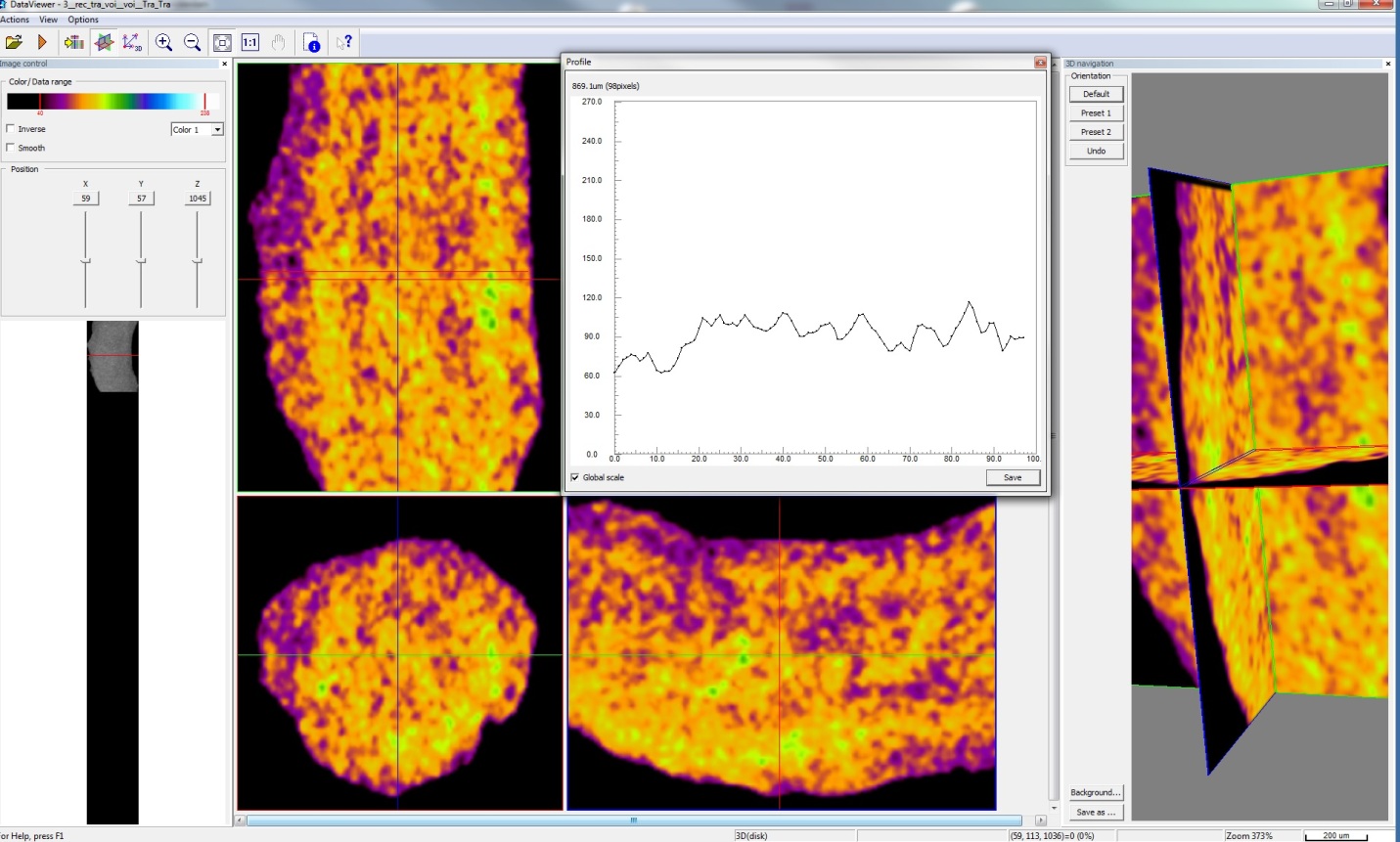


**Figure S5**. Analysis of X-ray contrast density and topology of the cross-section density of the root growth of sample D


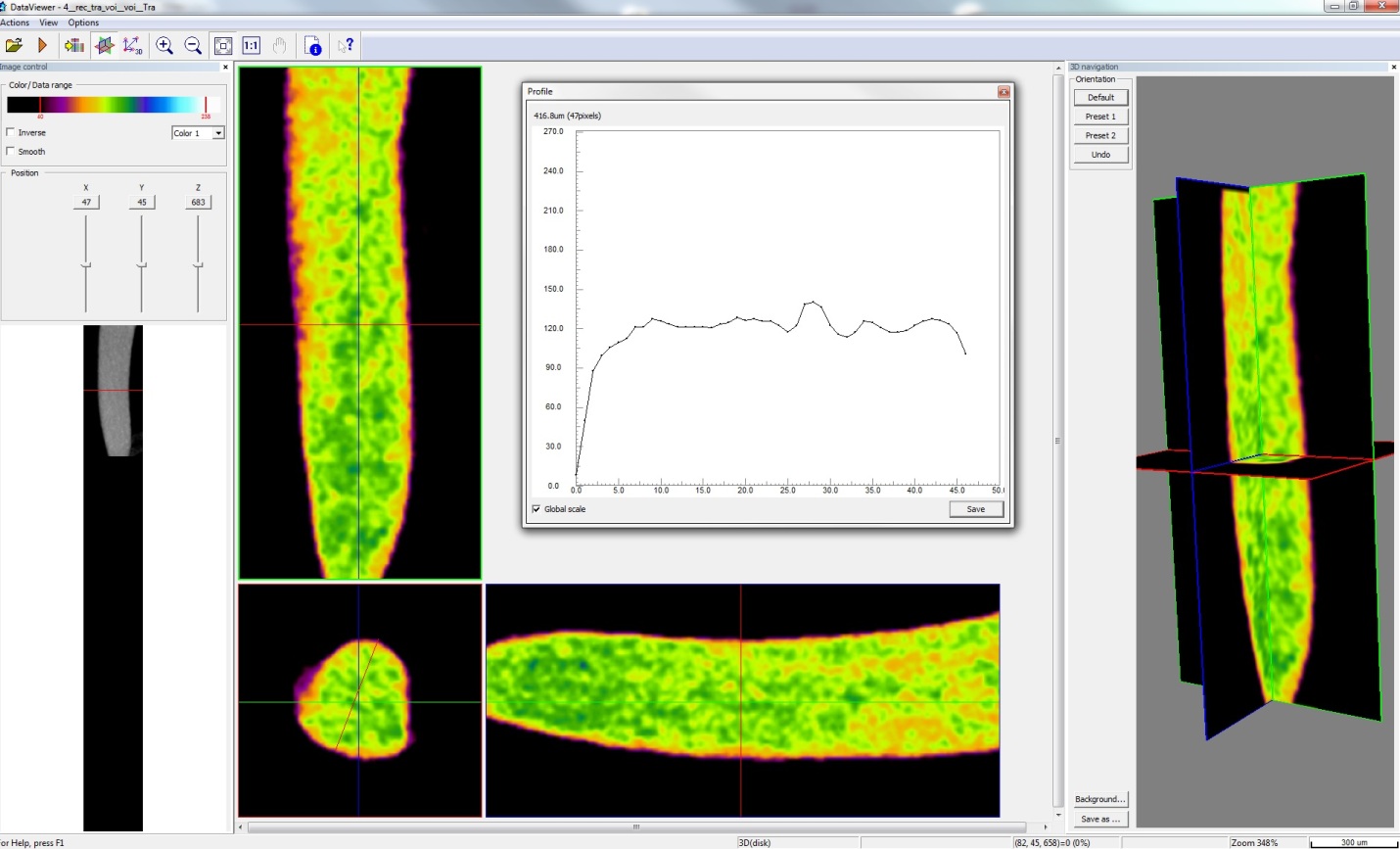


**Figure S6**. Analysis of X-ray contrast density and topology of the cross-section density of the root growth of sample E
